# Supplementary material for: Synthesis, Anticancer Activity and Molecular Docking Studies of Novel N-Mannich Bases of 1,3,4-Oxadiazole Based on 4,6-Dimethylpyridine Scaffold
Source: Int J Mol Sci. 2022 Sep 22;23(19):11173. doi: 10.3390/ijms231911173 (PMC9570134; doi:10.3390/ijms231911173)
Supplement: Supplementary file 1 [file ijms-23-11173-s001.zip › ijms-1897481-supplementary.pdf]

SUPPLEMENTARY MATERIALS

Synthesis, Anticancer Activity and Molecular Docking Studies of Novel *N*-Mannich Bases of 1,3,4-Oxadiazole Based on 4,6-Dimethylpyridine Scaffold

Małgorzata Strzelecka [1], Teresa Glomb [1,*], Małgorzata Drąg-Zalesińska [2], Julita Kulbacka [3], Anna Szewczyk [3,4], Jolanta Saczko [3], Paulina Kasperkiewicz-Wasilewska [5], Nina Rembiałkowska[3], Kamil Wojtkowiak[6], Aneta Jezierska [6] and Piotr Świątek [1,*]

[1] Department of Medicinal Chemistry, Faculty of Pharmacy, Wroclaw Medical University, Borowska 211, 50-556 Wroclaw, Poland
[2] Division of Histology and Embryology, Department of Human Morphology and Embryology, Faculty of Medicine, Wroclaw Medical University, Chałubińskiego 6a, 50-368 Wroclaw, Poland
[3] Department of Molecular and Cellular Biology, Faculty of Pharmacy, Wroclaw Medical University, Borowska 211, 50-556 Wroclaw, Poland
[4] Department of Animal Developmental Biology, Institute of Experimental Biology, University of Wroclaw, Sienkiewicza 21, 50-335 Wroclaw, Poland
[5] Department of Bioorganic Chemistry, Faculty of Chemistry, Wroclaw University of Science and Technology, Wybrzeże Wyspiańskiego 27, 50-370 Wroclaw, Poland
[6] Faculty of Chemistry, University of Wroclaw, F. Joliot-Curie 14, 50-383 Wroclaw, Poland

* Correspondence: piotr.swiatek@umw.edu.pl (P.Ś.); teresa.glomb@umw.edu.pl (T.G.); Tel.: +48-717840391

Table S1. NMR spectra of new compounds

**3**
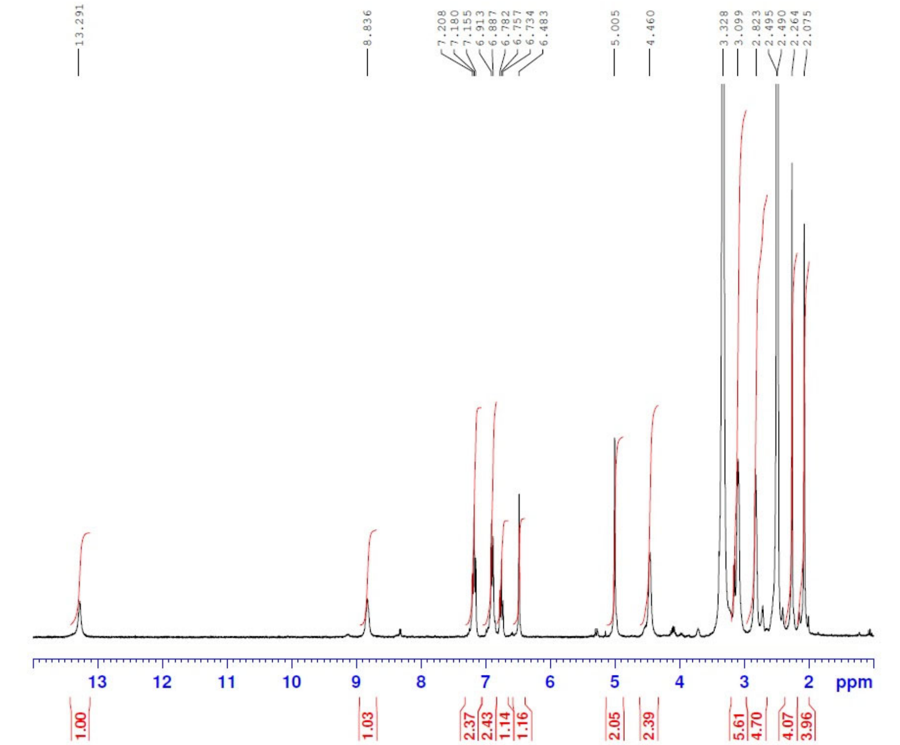
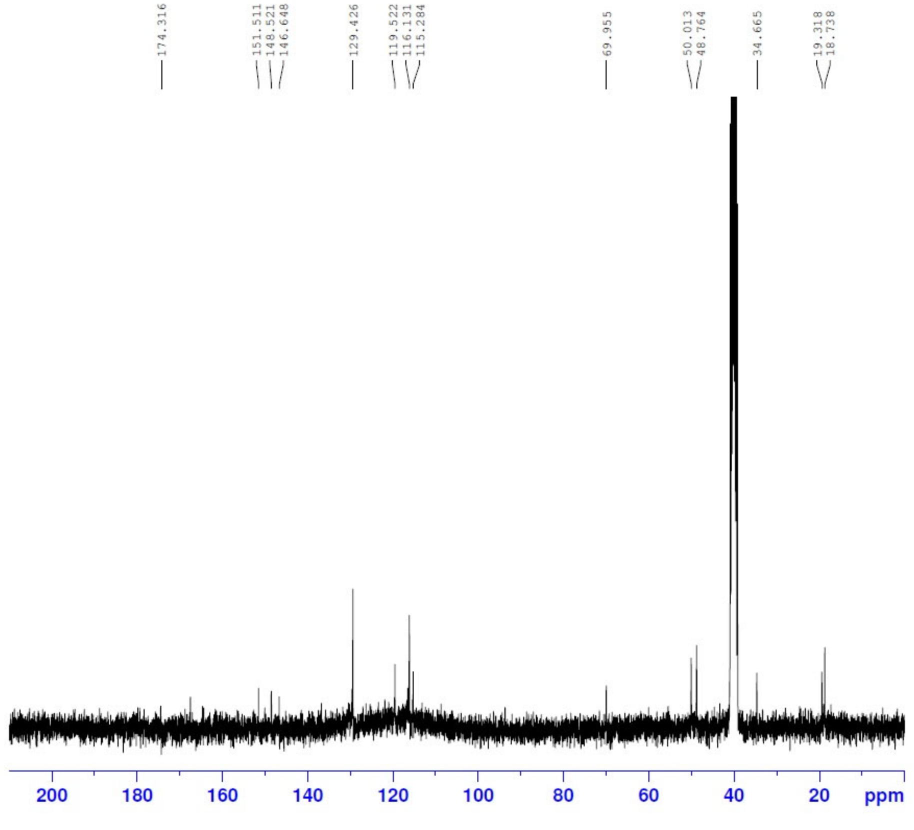

4

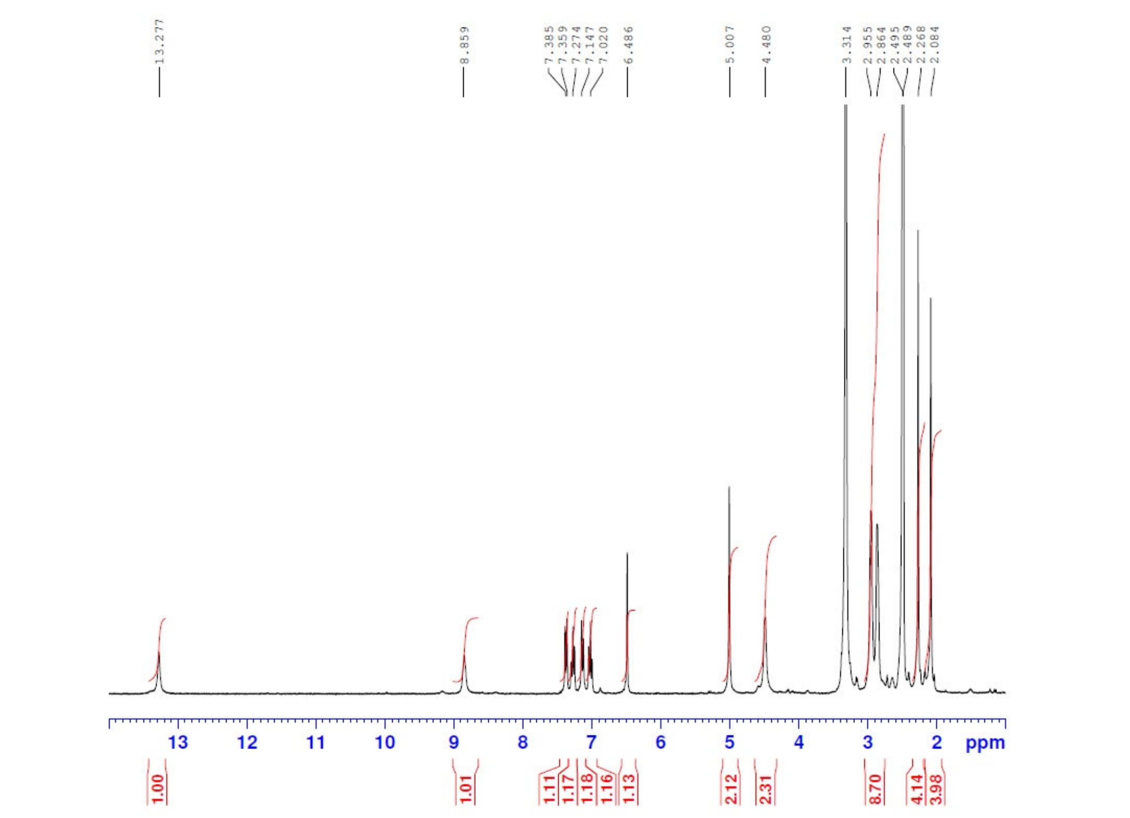
13.277
8.859
7.385 7.359 7.274 7.147 7.020
6.486
5.007
4.480
3.314
2.955 2.864 2.495 2.489 2.268 2.084
13 12 11 10 9 8 7 6 5 4 3 2 ppm
1.00
1.01
1.11 1.17 1.18 1.16 1.13
2.12
2.31
8.70
4.14 3.98

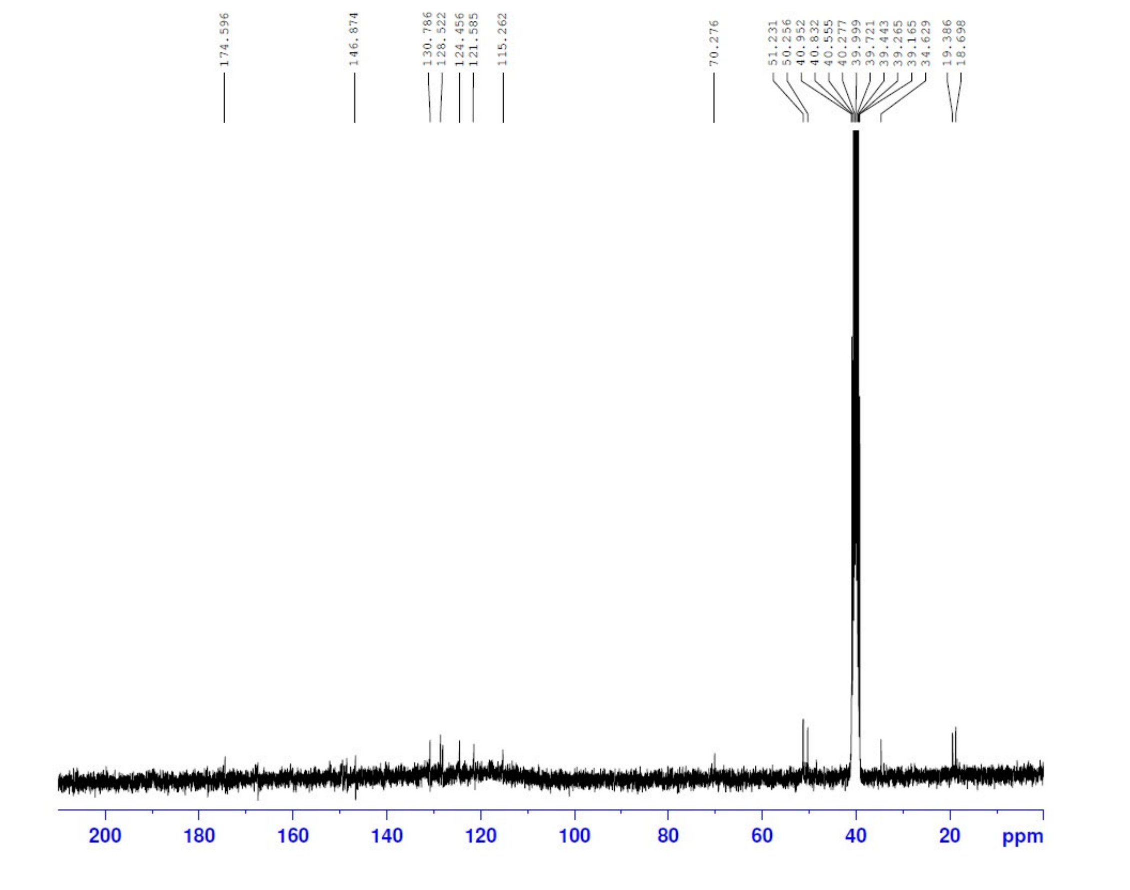
174.596
146.874
130.786 128.522 124.456 121.585
115.262
70.276
51.231 50.256 40.952 40.832 40.555 40.277 39.999 39.721 39.443 39.265 39.165 34.629
19.386 18.698
200 180 160 140 120 100 80 60 40 20 ppm

5

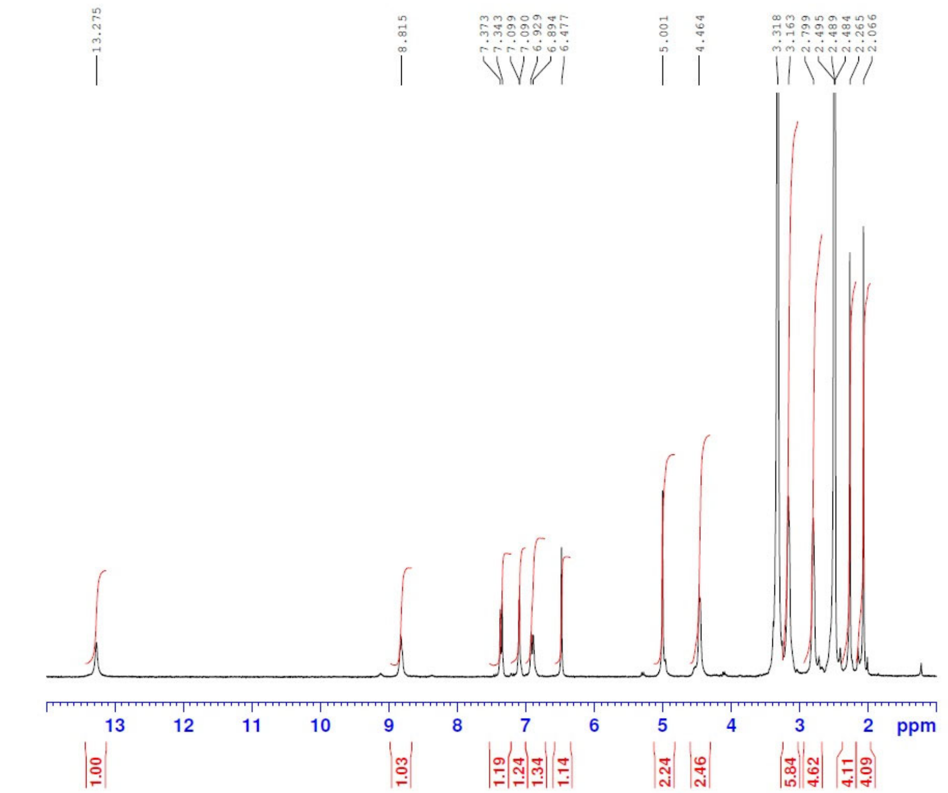
13.275
8.815
7.373
7.343
7.099
7.090
6.929
6.894
6.477
5.001
4.464
3.318
3.163
2.799
2.495
2.489
2.484
2.265
2.066
13
12
11
10
9
8
7
6
5
4
3
2
ppm
1.00
1.03
1.19
1.24
1.34
1.14
2.24
2.46
5.84
4.62
4.11
4.09

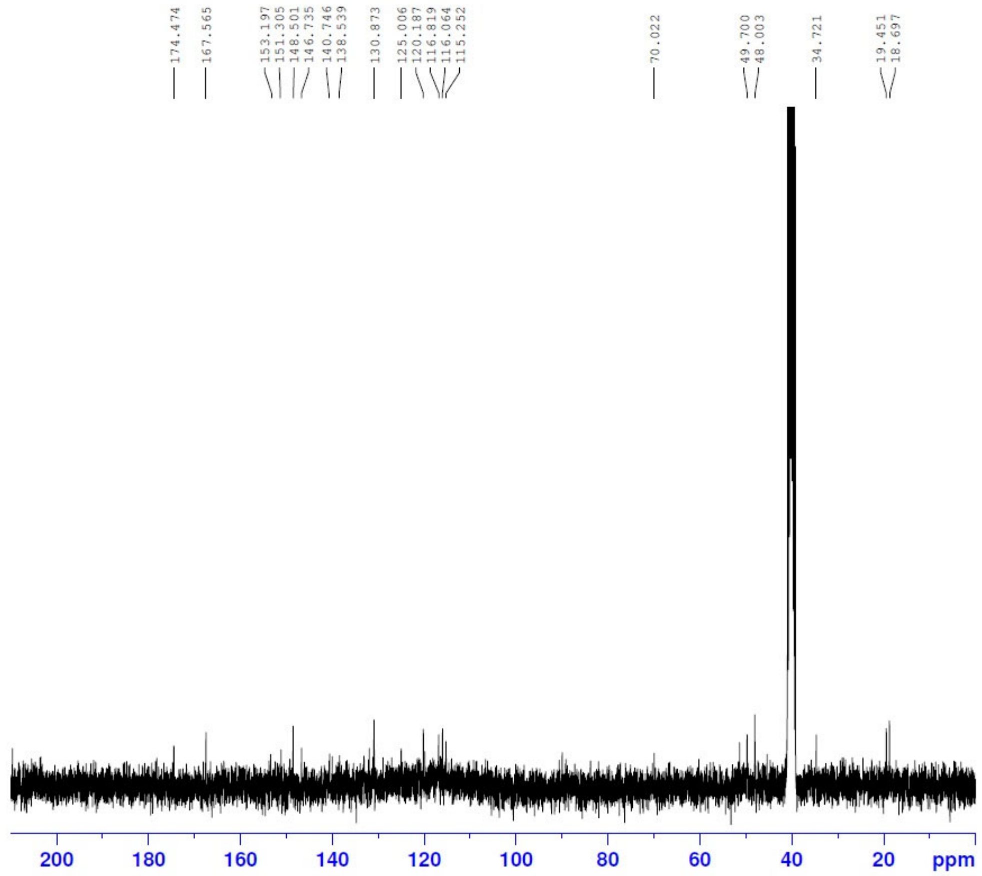
174.474
167.565
153.197
151.305
148.501
146.735
140.746
138.539
130.873
125.006
120.187
116.819
116.064
115.252
70.022
49.700
48.003
34.721
19.451
18.697
200
180
160
140
120
100
80
60
40
20
ppm

6

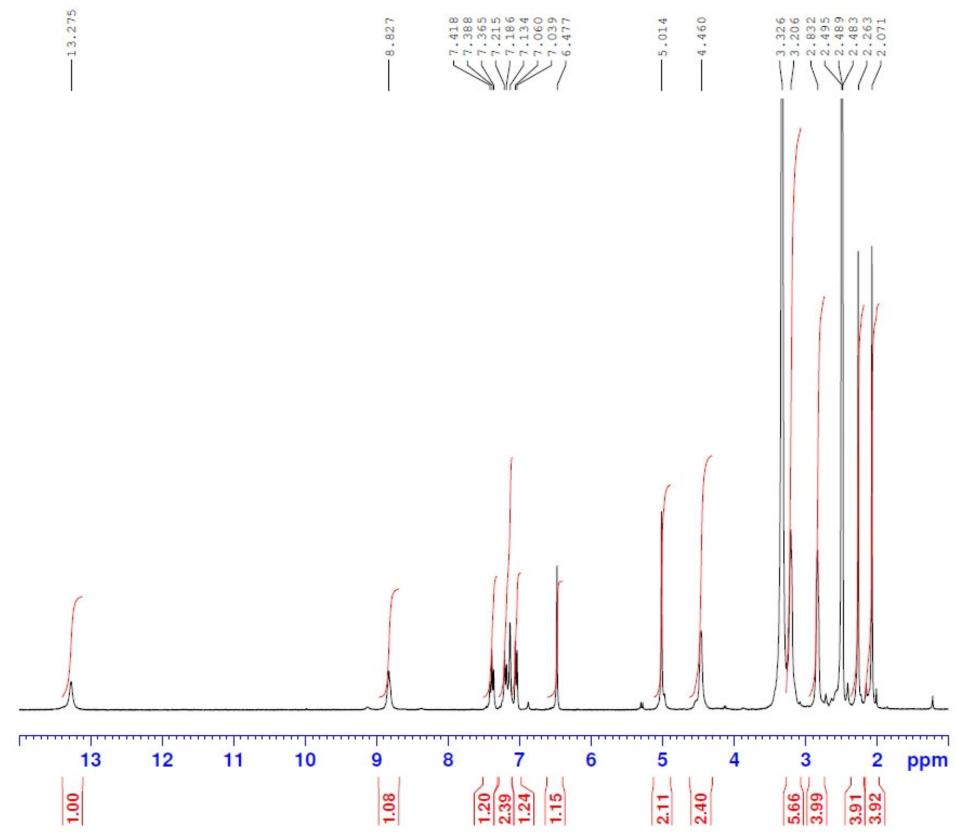
13.275
8.827
7.418
7.388
7.365
7.215
7.186
7.134
7.060
7.039
6.477
5.014
4.460
3.326
3.206
2.832
2.495
2.489
2.483
2.263
2.071
13
12
11
10
9
8
7
6
5
4
3
2
ppm
1.00
1.08
1.20
2.39
1.24
1.15
2.11
2.40
5.66
3.99
3.91
3.92

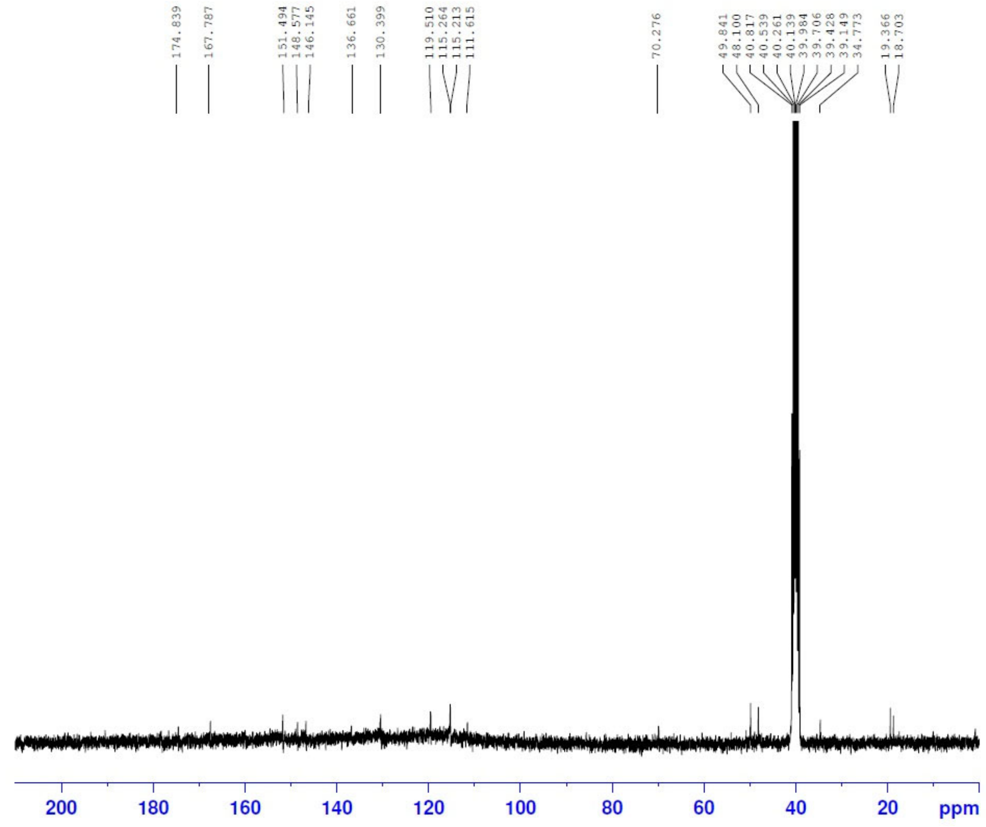
174.839
167.787
151.494
148.577
146.145
136.661
130.399
119.510
115.264
115.213
111.615
70.276
49.841
48.100
40.817
40.539
40.261
40.139
39.984
39.706
39.428
39.149
34.773
19.366
18.703
200
180
160
140
120
100
80
60
40
20
ppm

7

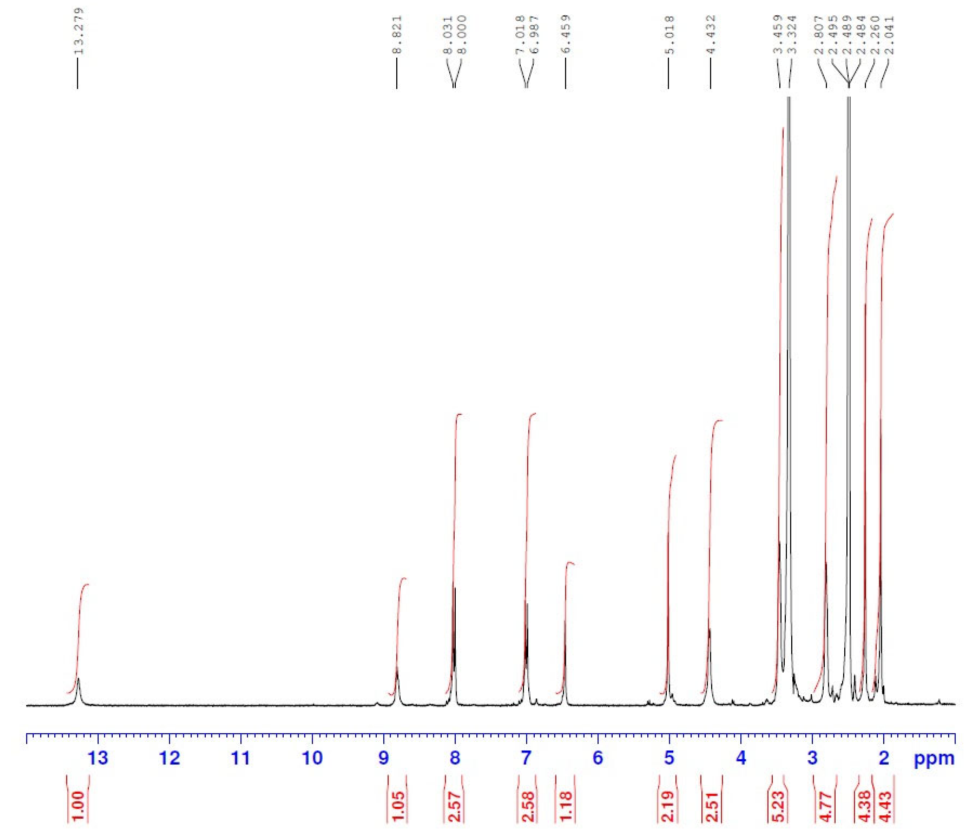
13.279
8.821
8.031
8.000
7.018
6.987
6.459
5.018
4.432
3.459
3.324
2.807
2.495
2.489
2.484
2.260
2.041
13
12
11
10
9
8
7
6
5
4
3
2
ppm
1.00
1.05
2.57
2.58
1.18
2.19
2.51
5.23
4.77
4.38
4.43

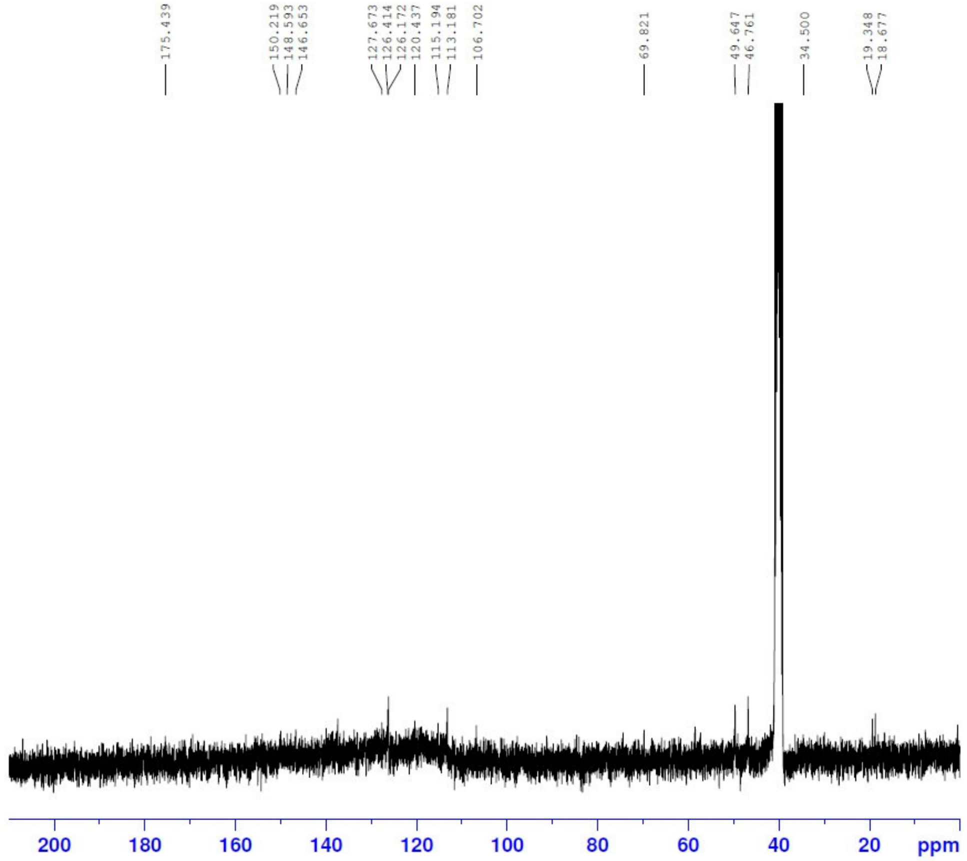
175.439
150.219
148.593
146.653
127.673
126.414
126.172
120.437
115.194
113.181
106.702
69.821
49.647
46.761
34.500
19.348
18.677
200
180
160
140
120
100
80
60
40
20
ppm

8

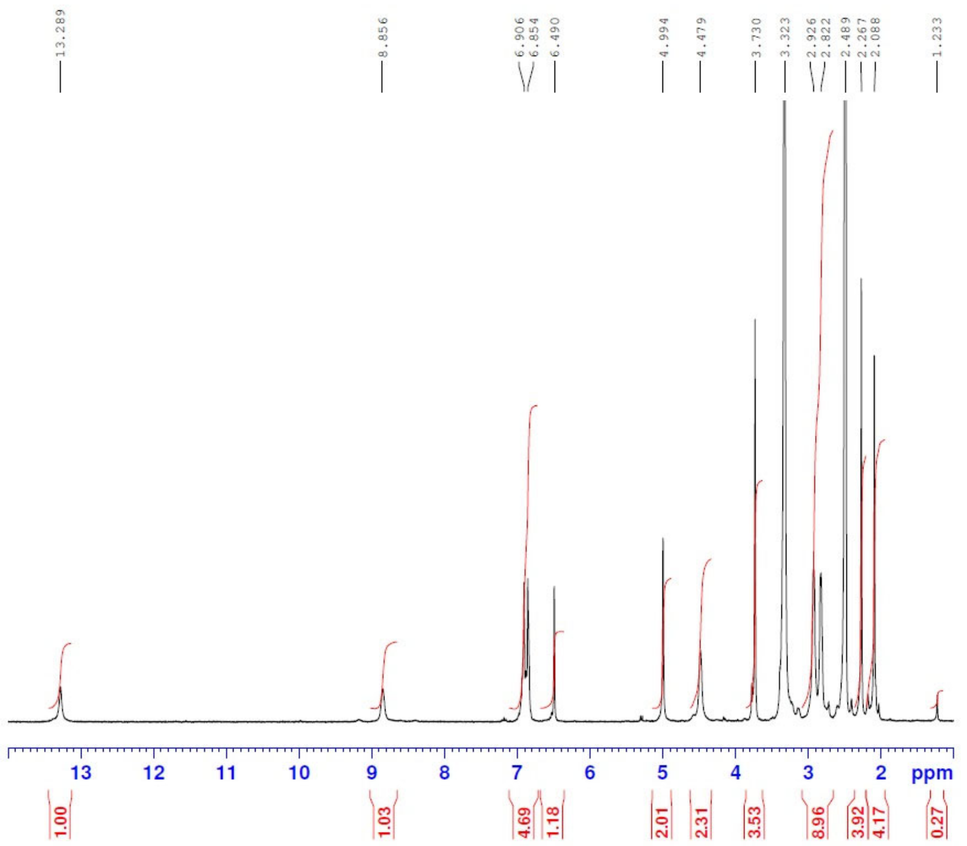
13.289
8.856
6.906
6.854
6.490
4.994
4.479
3.730
3.323
2.926
2.822
2.489
2.267
2.088
1.233
ppm
1.00
1.03
4.69
1.18
2.01
2.31
3.53
8.96
3.92
4.17
0.27

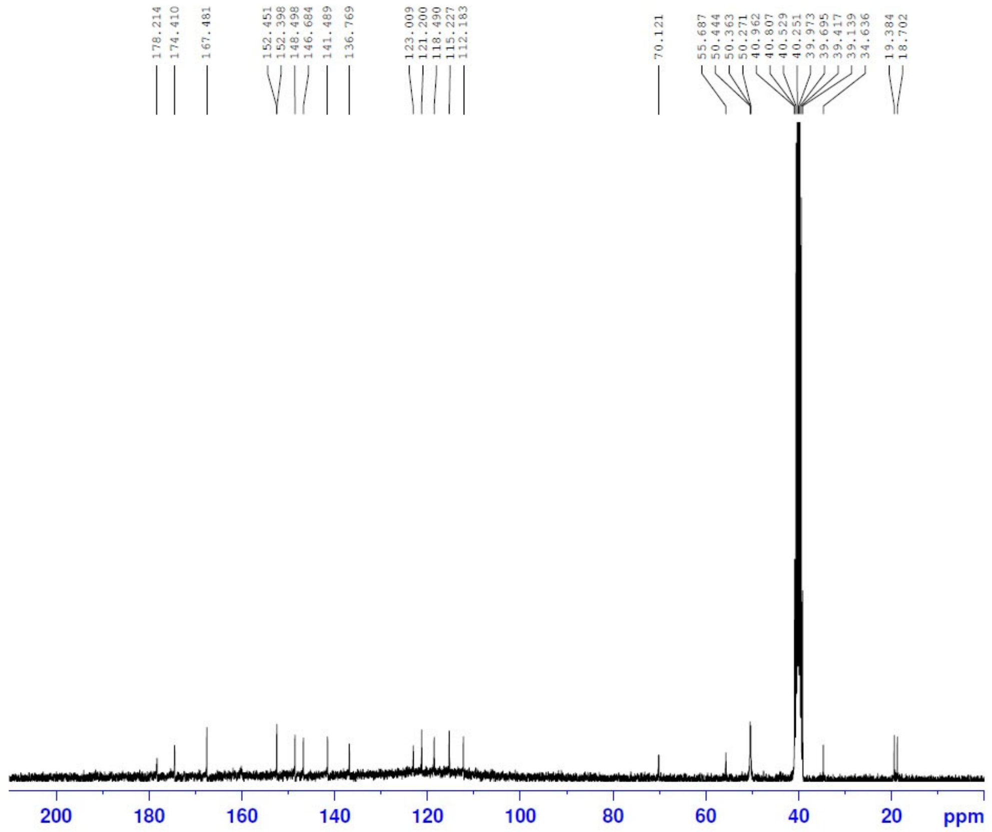
178.214
174.410
167.481
152.451
152.398
148.498
146.684
141.489
136.769
123.009
121.200
118.490
115.227
112.183
70.121
55.687
50.444
50.363
50.271
40.962
40.807
40.529
40.251
39.973
39.695
39.417
39.139
34.636
19.384
18.702
ppm

9

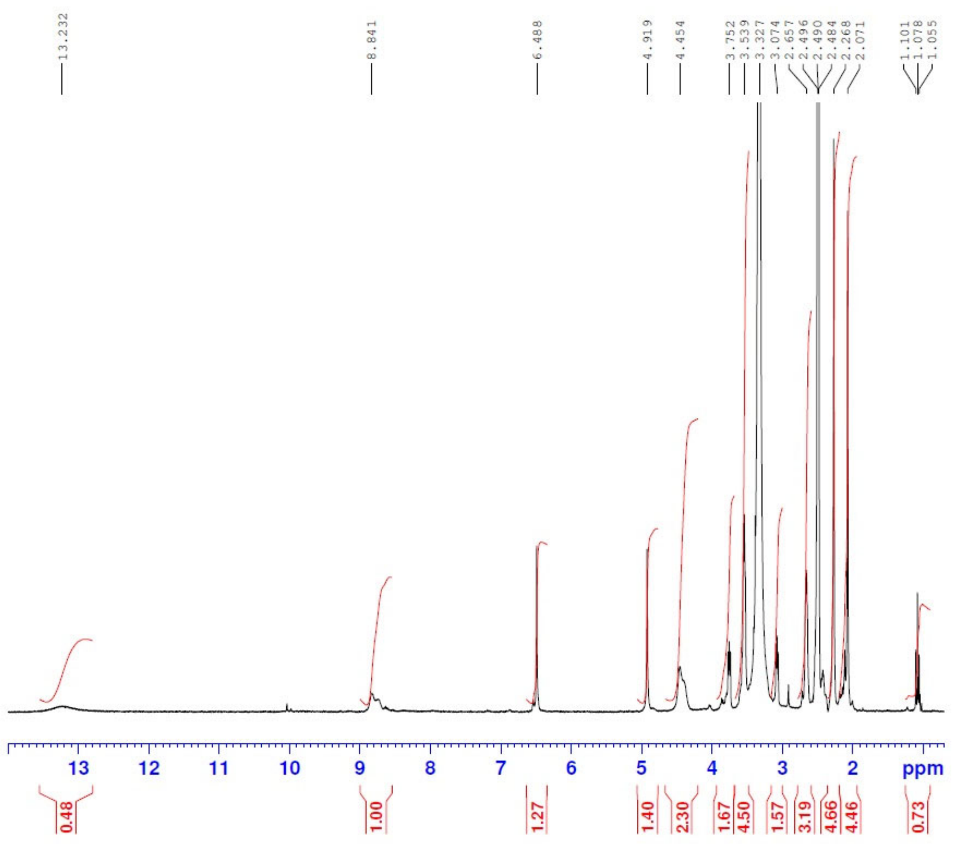
13.232
8.841
6.488
4.919
4.454
3.752
3.539
3.327
3.074
2.657
2.496
2.490
2.484
2.268
2.071
1.101
1.078
1.055
13 12 11 10 9 8 7 6 5 4 3 2 ppm
0.48
1.00
1.27
1.40
2.30
1.67
4.50
1.57
3.19
4.66
4.46
0.73

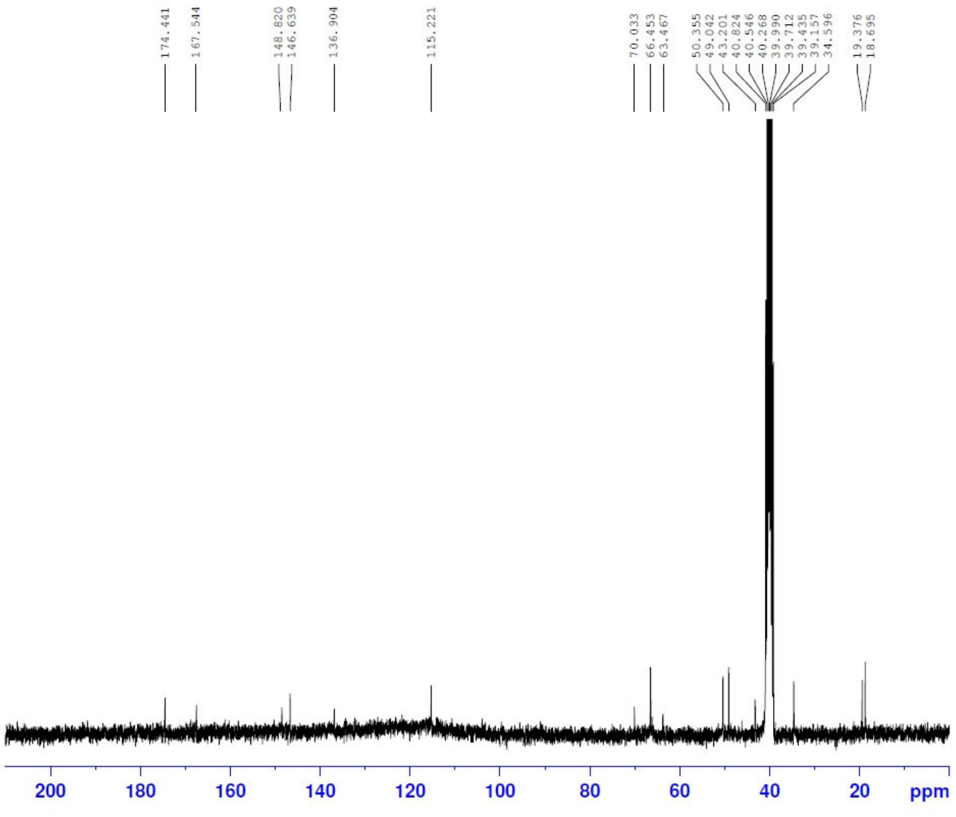
174.441
167.544
148.820
146.639
136.904
115.221
70.033
66.453
63.467
50.355
49.042
43.201
40.824
40.546
40.268
39.990
39.712
39.435
39.157
34.596
19.376
18.695
200 180 160 140 120 100 80 60 40 20 ppm

10

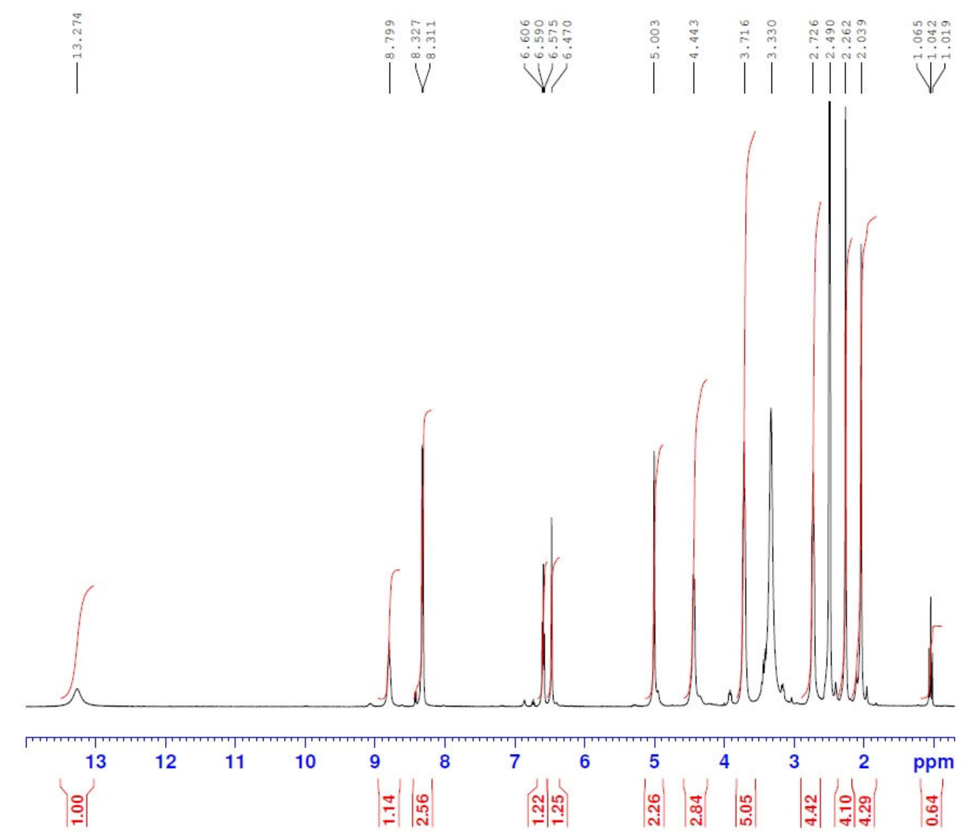
13.274
8.799
8.327
8.311
6.606
6.590
6.575
6.470
5.003
4.443
3.716
3.330
2.726
2.490
2.262
2.039
1.065
1.042
1.019
13 12 11 10 9 8 7 6 5 4 3 2 ppm
1.00
1.14
2.56
1.22
1.25
2.26
2.84
5.05
4.42
4.10
4.29
0.64

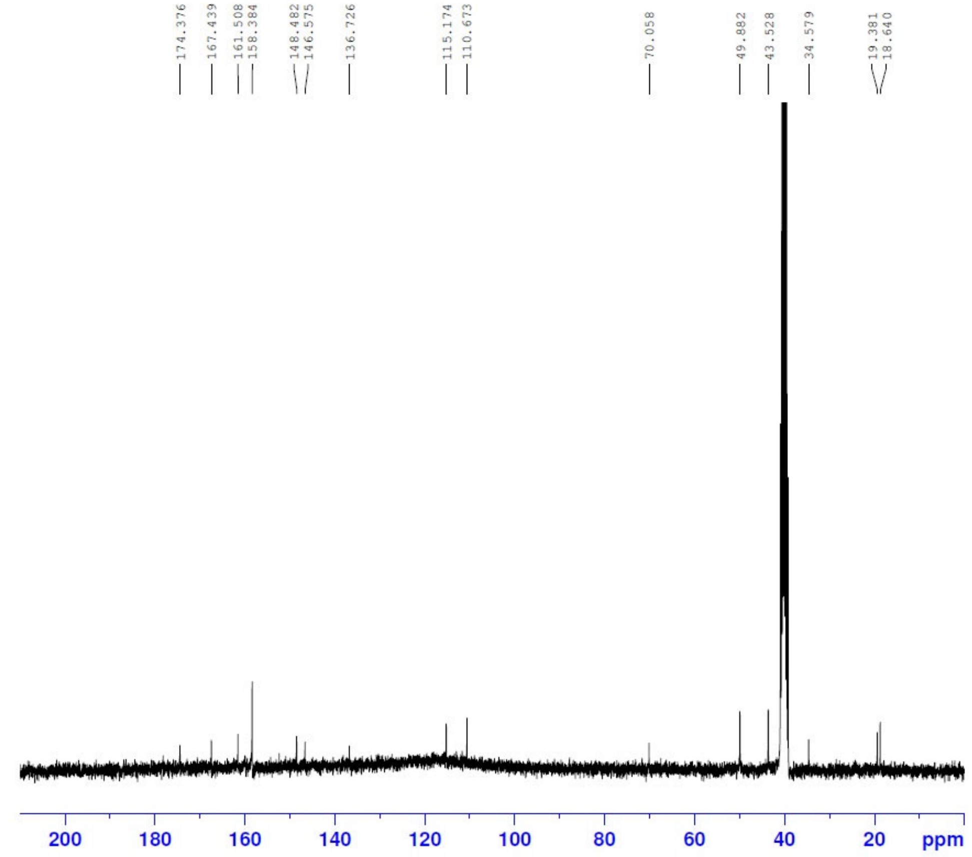
174.376
167.439
161.508
158.384
148.482
146.575
136.726
115.174
110.673
70.058
49.882
43.528
34.579
19.381
18.640
200 180 160 140 120 100 80 60 40 20 ppm

11

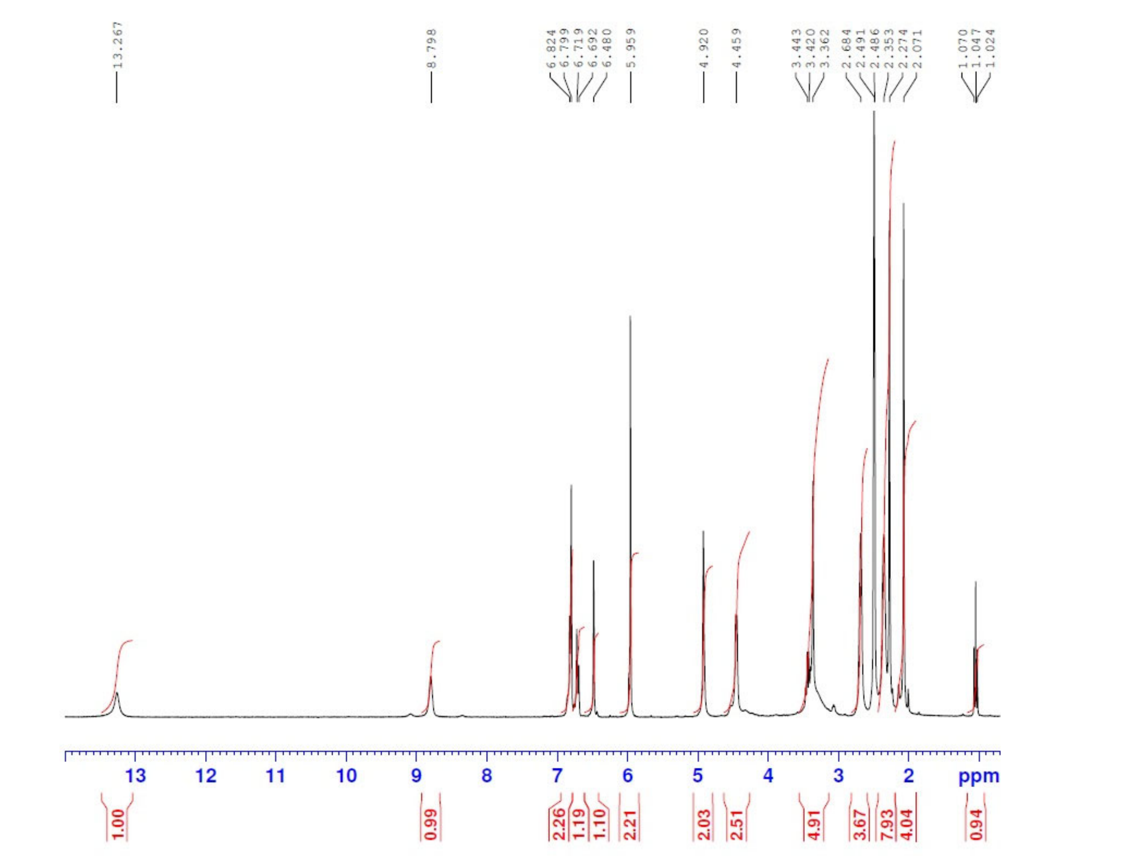
13.267
8.798
6.824
6.799
6.719
6.692
6.480
5.959
4.920
4.459
3.443
3.420
3.362
2.684
2.491
2.486
2.353
2.274
2.071
1.070
1.047
1.024
13 12 11 10 9 8 7 6 5 4 3 2 ppm
1.00
0.99
2.26
1.19
1.10
2.21
2.03
2.51
4.91
3.67
7.93
4.04
0.94

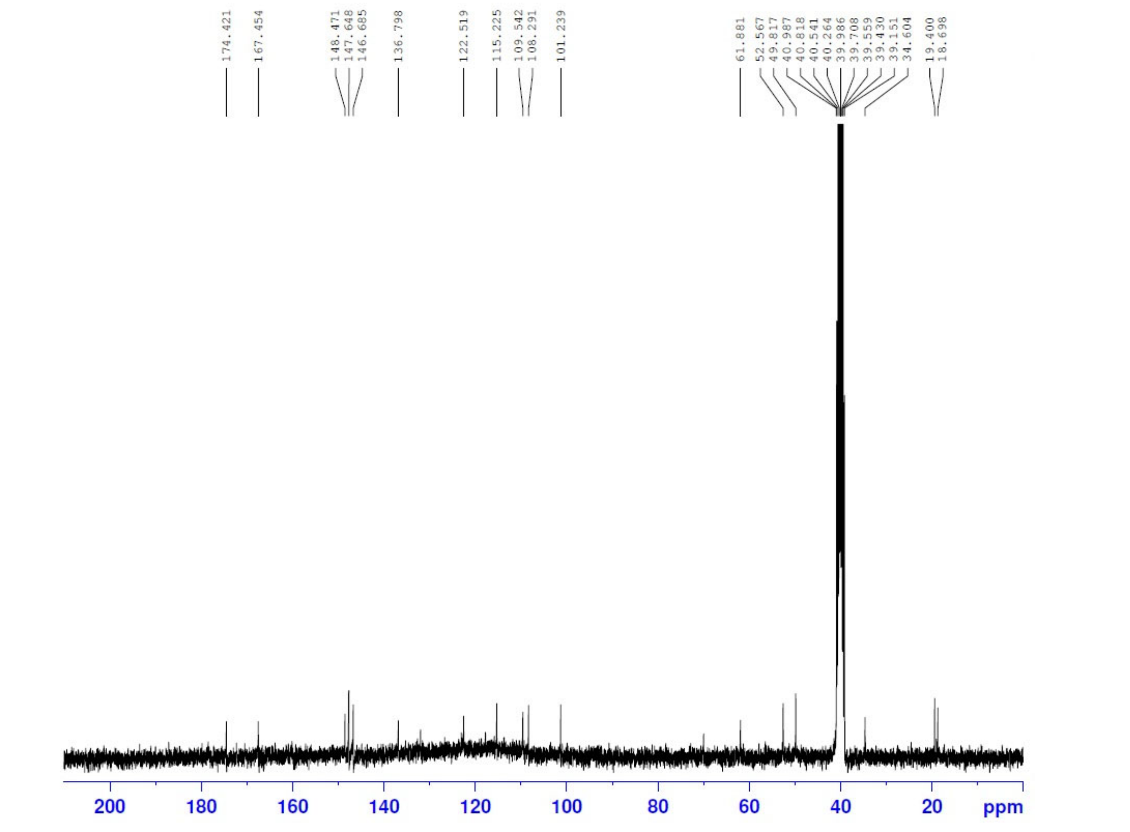
174.421
167.454
148.471
147.648
146.685
136.798
122.519
115.225
109.542
108.291
101.239
61.881
52.567
49.817
40.987
40.818
40.541
40.264
39.986
39.708
39.559
39.430
39.151
34.604
19.400
18.698
200 180 160 140 120 100 80 60 40 20 ppm

12

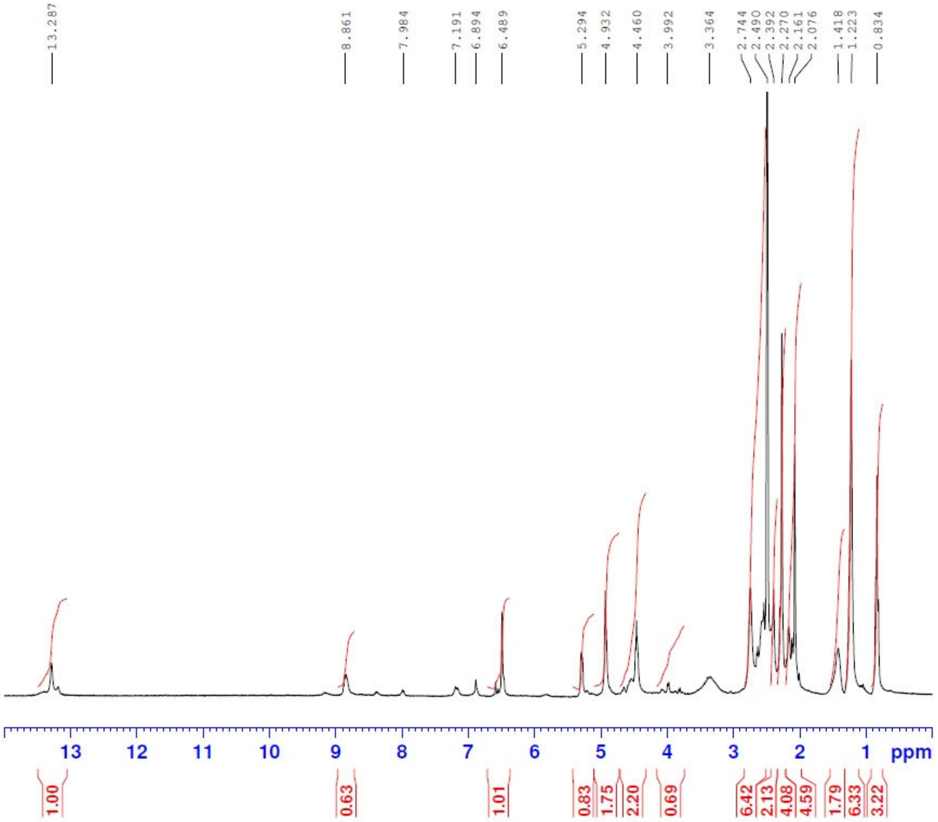

13.287
8.861
7.984
7.191
6.894
6.489
5.294
4.932
4.460
3.992
3.364
2.744
2.490
2.392
2.270
2.161
2.076
1.418
1.223
0.834
13 12 11 10 9 8 7 6 5 4 3 2 1 ppm
1.00
0.63
1.01
0.83
1.75
2.20
0.69
6.42
2.13
4.08
4.59
1.79
6.33
3.22

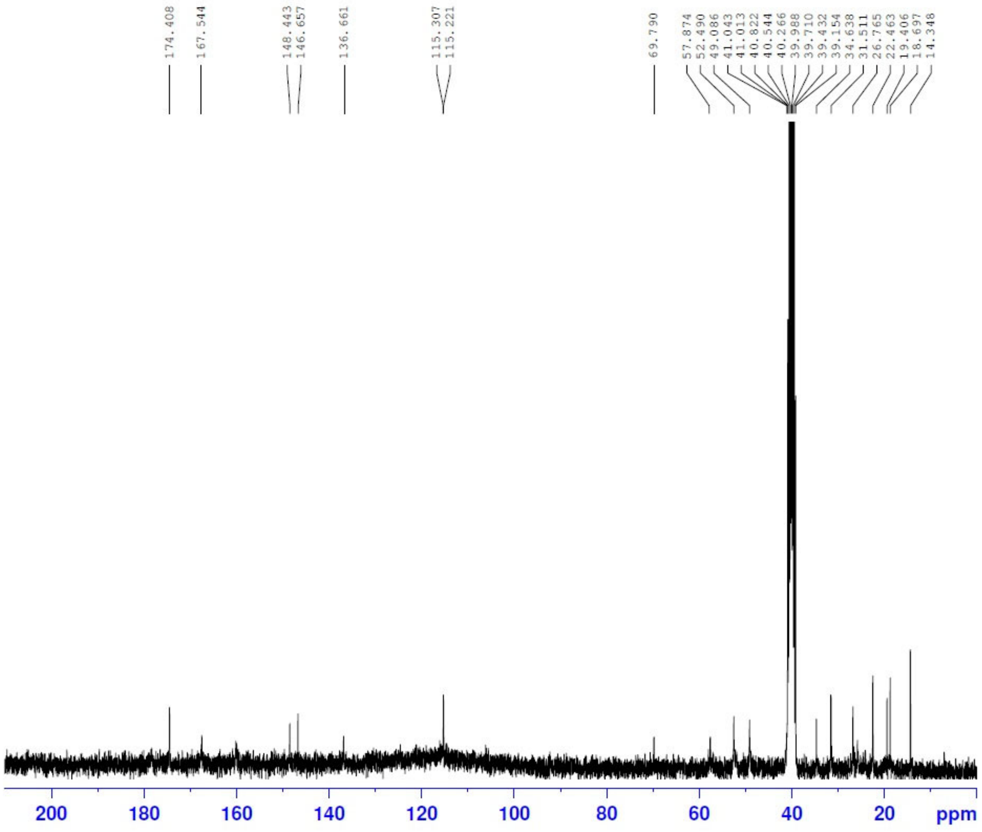

174.408
167.544
148.443
146.657
136.661
115.307
115.221
69.790
57.874
52.490
49.086
41.043
41.013
40.822
40.544
40.266
39.988
39.710
39.432
39.154
34.638
31.511
26.765
22.463
19.406
18.697
14.348
200 180 160 140 120 100 80 60 40 20 ppm
